# Effect of Secondary Phase on Passivation Layer of Super Duplex Stainless Steel UNS S 32750: Advanced Safety of Li-Ion Battery Case Materials

**DOI:** 10.3390/ma17112760

**Published:** 2024-06-05

**Authors:** Byung-Hyun Shin, Seongjun Kim, Jinyong Park, Jung-Woo Ok, Dohyung Kim, Jang-Hee Yoon

**Affiliations:** 1 Busan Centre, Korea Basic Science Institute, Busan 46742, Republic of Korea; lemonhouse211@kbsi.re.kr (B.-H.S.); seongjunk@kbsi.re.kr (S.K.); jinyongp@kbsi.re.kr (J.P.); jwok@kbsi.re.kr (J.-W.O.); 2 Innovative Graduate Education Program for Global High-Tech Materials and Parts, Pusan National University, Busan 46241, Republic of Korea

**Keywords:** super duplex stainless steel, passivation layer, chemical composition, secondary phase, Li-ion battery case materials, oxide layer depth

## Abstract

Aluminum, traditionally the primary material for battery casings, is increasingly being replaced by UNS S 30400 for enhanced safety. UNS S 30400 offers superior strength and corrosion resistance compared to aluminum; however, it undergoes a phase transformation owing to stress during processing and a lower high-temperature strength. Duplex stainless steel UNS S 32750, consisting of both austenite and ferrite phases, exhibits excellent strength and corrosion resistance. However, it also precipitates secondary phases at high temperatures, which are known to form through the segregation of Cr and Mo. Various studies have investigated the corrosion resistance of UNS S 32750; however, discrepancies exist regarding the formation and thickness of the passivation layer. This study analyzed the oxygen layer on the surface of UNS S 32750 after secondary-phase precipitation. The microstructure, volume fraction, chemical composition, and depth of O after the precipitation of the secondary phases in UNS S 32750 was examined using FE-SEM, EDS, EPMA and XRD, and the surface chemical composition and passivation layer thickness were analyzed using electron probe microanalysis and glow-discharge spectroscopy. This study demonstrated the segregation of alloy elements and a reduction in the passivation-layer thickness after precipitation from 25 μm to 20 μm. The findings of the analysis aid in elucidating the impact of secondary-phase precipitation on the passivation layer.

## 1. Introduction

The demand for Li-ion batteries has been steadily increasing owing to the increasing use of electric vehicles and portable electronic devices [1,2]. Given the risk of explosions due to battery overheating during use, considerable research has been conducted to ensure the safety of these batteries [3,4]. Rahman studied the monitoring system and Petit studied the battery lifetime [5,6]. Systems and catalysts that suppress heat generation have been explored to control overheating. Rahman designed a system to monitor the temperature of battery systems for heat control and Klink developed sensor- and model-based thermal runaway techniques [6,7]. These studies aimed to detect the state of the battery in the early stages of heat generation; however, delaying the reaction was inevitable once the heating reaction began [8,9]. Koelva studied the hybrid battery storage [10]. Therefore, systems have been developed to suppress explosions at high temperatures during heating reactions. However, the fundamental issues of safety improvements at high temperatures remain unresolved. Consequently, recent innovations have been presented regarding the materials used for Li-ion battery casings. The casing material was upgraded from aluminum to UNS S 30400, which offers higher strength and corrosion resistance, thereby significantly enhancing the safety of Li-ion batteries.

UNS S 30400, an austenite stainless steel, exhibits excellent strength and corrosion resistance [11,12,13]. However, its strength and corrosion resistance are lower during processing because of its low high-temperature strength (170 MPa at 700 °C) and martensite transformation caused by stress (difference in lattice structure with austenite and reduced corrosion resistance) [14,15,16]. The low high-temperature strength is because of the high alloy content and unstable face-centered cubic (FCC) structure. A stress-induced martensite (ε-martensite, base-centered tetragonal (BCT)) transformation occurs when the unstable austenite, cooled after a solution heat treatment, is subjected to stress. Martensite, being a BCT structure, has fewer slip systems, which contributes to a higher strength but makes it susceptible to impact and lowers its corrosion resistance. Additionally, the high thermal-expansion coefficient of UNS S 30400 (16.6 × 10^−6^ ppm/°C) accelerates corrosion and decreases the high-temperature strength at elevated temperatures. These characteristics were extensively studied and gained attention in the early 1990s. Thus, UNS S 30400, which is one of the most renowned materials, can be easily applied in various fields. However, recently developed materials with superior properties are suitable replacements for UNS S 30400.

Duplex stainless steel is an appropriate replacement for UNS S 30400 owing to its high corrosion resistance and high-temperature strength [17,18,19]. Duplex stainless steel demonstrates superior strength and corrosion resistance owing to its phase of austenite and ferrite. Among duplex stainless steels, the super duplex stainless steel (SDSS) with a pitting resistance equivalent number (PREN) of 42 showcases outstanding strength and corrosion resistance. It also maintains high-temperature strength exceeding 300 MPa at 700 °C and a low thermal-expansion coefficient (13.5 × 10^−6^ ppm/°C) [20,21,22]. These attributes significantly enhance the safety of Li-ion battery casings. Although extensively researched for welding applications, primarily in recent years, the processing of SDSS induces the precipitation of secondary phases, altering its physical and electrochemical properties [22,23,24]. Therefore, research on the characteristics of these secondary phases is necessary, particularly concerning their impact on the passivation layers. However, studies on the effects of secondary-phase precipitation on the characteristics of the passivation layer have been scarce [25,26,27].

UNS S 32750, a major grade representing SDSS, exhibits high-temperature strength. However, the high chemical composition of 25 wt% Cr and 3.8 wt% Mo makes it prone to the precipitation of secondary phases (embrittlement and decreased corrosion resistance) [28,29,30]. The ignition temperature of Li-ion batteries, 700 °C, triggers the precipitation of these secondary phases in UNS S 32750. This leads to alloy segregation due to the precipitation of Cr and Mo, resulting in reduced corrosion resistance and high-temperature cracking. Nillson focused on the corrosion resistance of UNS S 32750 with respect to heat-treatment temperatures and analyzed the corrosion resistance after the precipitation of secondary phases [29]. Similarly, Amatsuka analyzed the phase-fraction changes and corrosion resistance of UNS S 32750 based on heat-treatment temperatures and chemical compositions by employing corrosion-resistance analysis based on the volume fractions of phase [31]. Martins analyzed the microstructure of cast UNS S 32750. While many studies have investigated the microstructure and corrosion resistance of UNS S 32750, research on the precipitation of secondary phases at 700 °C and its effect on the passivation layer remains scarce. Therefore, further research is necessary to elucidate the changes in the passivation layer after the phase transformation occurring at approximately 700 °C in order to achieve the effective application of UNS S 32750 in Li-ion battery casings.

This study aimed to investigate the influence of the precipitation of the secondary phase on the passivation layer by examining the precipitation of secondary phases at 700 °C after heat treatment and analyzing the state of the passivation layer accordingly. UNS S 32750 was produced by casting, followed by heat treatments at 1100 °C for solution annealing and 700 °C to simulate the ignition temperature of Li-ion batteries. The morphology and precipitation patterns of the secondary phases after the heat treatment at the ignition temperature of the Li-ion batteries were examined using field-emission scanning electron microscopy (FE-SEM), electron backscatter diffraction (EBSD), and X-ray diffraction (XRD). Factors affecting the passivation layer were analyzed using electron-probe microanalysis (EPMA), glow-discharge spectroscopy (GDS), and X-ray photoelectron spectroscopy (XPS). EPMA was used to analyze the alloy distribution, and GDS was used to measure the depth of the passivation layer.

## 2. Experimental

### 2.1. Materials

The material used in this study—UNS S 32750—was classified as SDSS [16,29,32]. The chemical composition of UNS S 32750 cast in an electric furnace was analyzed using inductively coupled plasma mass spectrometry (ICP-MS; Thermo Fisher Scientific, Waltham, MA, USA), and the composition is presented in Table 1. The PREN for UNS S 32750 was calculated according to Equation (1) based on the chemical composition of its main alloys and had a value of 42.

Stainless steel is an alloy containing at least 12 wt% Cr, which forms a passivation layer of Cr_2_O_3_ that inhibits electrochemical reactions, thereby enhancing corrosion resistance [25,26,27,33]. Cr acts as the primary component of Cr_2_O_3_, whereas Mo, which has a larger lattice size than iron, induces stress in the Cr-defected zone, facilitating the formation of the passivation layer. N acts as an interstitial element, strengthening the corrosion resistance of void spaces within the passivation layer. The PREN was calculated according to the following formula:

PREN = wt% Cr + 3.3 wt% Mo + 16 wt% N(1)

### 2.2. Heat Treatment

**Figure 1:**
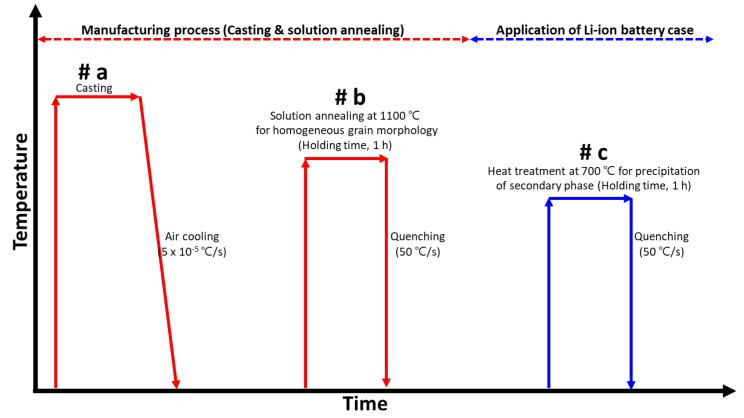
Schematic diagram of heat-treatment conditions for application on a Li-ion battery case of super duplex stainless steel UNS S 32750.

SDSS UNS S 32750 exhibited variations in phase distribution and composition depending on the heat-treatment conditions, making heat treatment a crucial factor in SDSS processing [34,35,36]. A schematic of the heat-treatment conditions employed in this study is shown in Figure 1. Casting (#a), followed by rapid cooling from high temperatures, facilitated the observation of the phase. UNS S 32750 was heat-treated in a box furnace. By comparing the microstructure after casting with that after the solution heat treatment, the grain-growth behaviors of austenite and ferrite were determined [18]. The solution heat treatment (#b, conducted at 1100 °C) yielded the most superior corrosion resistance as it equalized the volume fractions of austenite, ferrite, and the PREN [37,38]. Additionally, the uniform shape of austenite prevented high-temperature cracking induced by the secondary phases. Heat treatment was conducted at the ignition temperature (#c) of Li-ion batteries, 700 °C, to investigate the impact of secondary phases during the ignition of Li-ion batteries on the passivation layer. The samples were subsequently heat-treated at 700 °C for 5 h and cooled at a rate of 50 °C/s to observe the phase changes during the ignition of Li-ion batteries.

### 2.3. Microstructure and Phase

The microstructures formed under the heat-treatment conditions shown in Figure 1 were examined using an FE-SEM (SUPRA 40VP, Zeiss, Oberkochen, Germany) and EBSD (SUPRA 40VP, Zeiss, Oberkochen, Germany). The surface was polished using an abrasive paper and colloidal silica for microstructural examination after the heat treatment. Subsequently, electrolytic etching was performed at 5 V for 30 s in 10 wt% NaOH electrolyte solution [16,32,39]. The nonuniform microstructure observed after casting was homogenized by the solution heat treatment. The heat treatment was conducted at 700 °C, and the precipitation morphology of the secondary phase was examined thereafter. The red line in Figure 1 represents the typical manufacturing process of SDSS UNS S 32750. In this study, the conditions for the precipitation of the secondary phases, indicated by the blue line in Figure 1, were used to investigate the crystallization behavior of the secondary phases. The blue line in Figure 1 represents the conditions for examining the microstructure of Li-ion batteries, where the heat treatment was performed at 700 °C to observe the precipitation behavior of the secondary phases. The precipitation phases were identified using XRD (D8 VENTURE, Stanford, CA, USA) by confirming the 2θ intensity.

The volume fractions of the phases in UNS S 32750 were determined according to ASTM E 1245 [40]. The volume fraction was checked seven times, and the average of the mid-ranges was used.

### 2.4. Chemical Composition

After casting, SDSS UNS S 32750 exhibited a microstructure characterized by nonuniform austenite formation, with secondary phases forming at the austenite grain boundaries. The composition of SAF2507 was analyzed according to the heat-treatment conditions shown in Figure 1 by using energy-dispersive X-ray spectroscopy (EDS; SUPRA 40VP, Zeiss, Oberkochen, Germany) and EPMA (JXA-8530F, Tokyo, Japan). The compositions after casting and solution heat treatment are presented in Table 2. Differences in the chemical compositions, particularly those of Cr and Mo, were observed between the austenite and ferrite, on average, 5 times. Chemical-composition analysis revealed that austenite exhibited higher concentrations of FCC-stabilizing elements such as Ni, Mn, and N [41,42,43]. In contrast, ferrite showed a higher chemical composition of BCC-stabilizing elements such as Cr and Mo.

N plays a crucial role in enhancing the corrosion resistance of SDSS. However, because N is an interstitial element, the EDS analysis of N tends to be inaccurate. Therefore, the following formula was used for the calculations:

N*r* = Chemical composition of N total wt% − Ferrite*VF* × 0.05 wt%. (2)

The maximum N solubility in ferrite was 0.05 wt%, and the remaining N was dissolved in the austenite. Despite having lower Cr and Mo contents, austenite maintained an excellent passivation layer because of its higher N solubility compared to ferrite. In contrast, ferrite strengthened the passivation layer through Cr and Mo. Changes in the composition after the solution heat treatment were reflected in the alteration of the PRE, which decreased after casting. SDSS UNS S 32750 produced in this manner is machinable as a precursor for Li-ion batteries. Compositional changes were examined at 700 °C to ensure safety in case of ignition when SDSS UNS S 32750 is used as a material for Li-ion battery cases.

In addition, GDS (Horiba Jobin Yvon, JY-10000 RF, Edouard Belin Longjumeau, France) was conducted to examine the thickness and the chemical composition of the passivation layer as it enables the analysis of the chemical compositional changes along the depth direction. The changes in the chemical compositions of O, Cr, and Fe with depth were measured, and the results were verified. Therefore, the depth of the passivation layer was confirmed based on changes in the surface chemical composition observed using GDS.

### 2.5. Electrochemical Analysis Behavior

To check the status of the passivation layer, a potentiodynamic polarization test was conducted. The analysis was performed using a three-electrode cell and a potentiometer (Potentiostat, Versa Stat 4.0, AMETEK, Inc., Berwyn, PA, USA). The three-electrode cell consisted of the specimen, a reference electrode, and a counter electrode. The reference electrode used was a saturated calomel electrode (KCl electrolyte solution), and the counter electrode was a platinum mesh (20 × 20 mm). The potentiodynamic polarization test measured the change in current density with respect to voltage, and it was analyzed from −0.6 V to 1.2 V. According to ASTM G61 [44], a 3.5 wt% NaCl electrolyte solution was used, and the scan rate was 0.167 mV/s.

## 3. Results

### 3.1. Effect of Manufacturing Process

**Figure 2:**
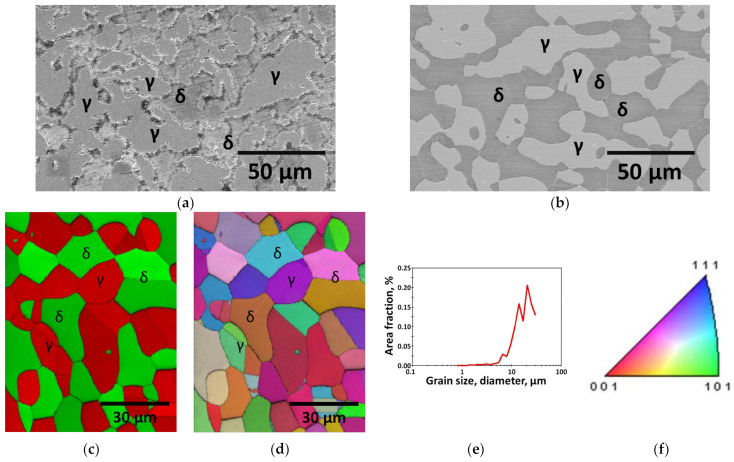
Microstructure image of super duplex stainless steel UNS S 32750: (**a**) cast and (**b**) solution annealed at 1100 °C (γ: austenite, and δ: ferrite), (**c**) Phase-IQ after solution annealing at 1100 °C, (**d**) IPF-IQ image after solution annealing at 1100 °C, (**e**) grain size and area fraction after solution annealing at 1100 °C, and (**f**) IPF color palette.

**Figure 3:**
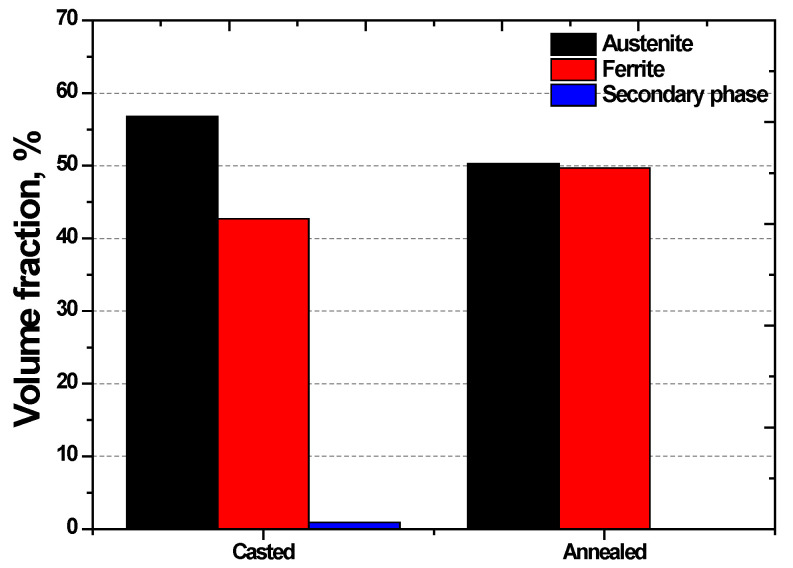
Volume fractions of austenite and ferrite for casting and solution-annealing manufacturing processes of super duplex stainless steel UNS S 32750.

UNS S 32750 was cast by adjusting its chemical composition in an electric furnace because of its high alloy contents. The casting was carried out at temperatures exceeding 2000 °C, leading to a phase transformation from liquid to solid [39]. Owing to the differences in cooling rates between the interior and exterior of the casting, the molten metal was poured into a mold and air-cooled to prevent high-temperature cracking. The microstructure after casting is shown in Figure 2a; that after casting and subsequent annealing is shown in Figure 2b–e, and Figure 2f is the IPF color palette of EBSD [26]. Figure 2c,d shown the phase-IQ and IPF-IQ. It shows the homogenized microstructure after heat treatment, as well as the uniform morphology and crystal direction. The volume fractions of phases based on this manufacturing process are shown in Figure 3 [18,29,39]. Cast UNS S 32750 formed an unevenly shaped austenite and precipitated the secondary phase. After annealing at 1100 °C, the microstructure of UNS S 32750 became more uniform, with an equal distribution of austenite and ferrite, resulting in the most corrosion-resistant condition with a 5:5 ratio [16,22,45].

The homogenized microstructure after solution treatment was confirmed using FE-SEM and EBSD. The grain size after solution treatment ranged from 20 to 30 μm, which is consistent with the grain sizes reported in the literature for similar solution treatments [16,29].

Table 2 lists the chemical compositions of the cast and annealed UNS S 32750. The chemical compositions of austenite and ferrite differed in UNS S 32750 after casting. These differences in the chemical composition based on the phase resulted in variations in the PRE. After annealing, the chemical compositions of the austenite and ferrite in UNS S 32750 still differed. Austenite contained 7.9 wt% Ni, 1.1 wt% Mn, and 0.51 wt% N, whereas ferrite had 26.6 wt% Cr and 4.4 wt% Mo. These variations in the chemical composition were attributed to the stabilizing elements specific to the lattice structure of each phase.

The PREN values of the cast and annealed UNS S 32750 samples differed. The uneven structure after casting resulted in differences in the proportion of austenite, which led to variations in the PRE. After solution annealing, the homogenized structure achieved equal proportions of austenite and ferrite, thereby equalizing the PRE. The uniformity of the structure and the equalized PREN indicate the state with the least amount of alloy precipitation, demonstrating a stabilized microstructure.

### 3.2. Precipitation of Secondary Phase

**Figure 4:**
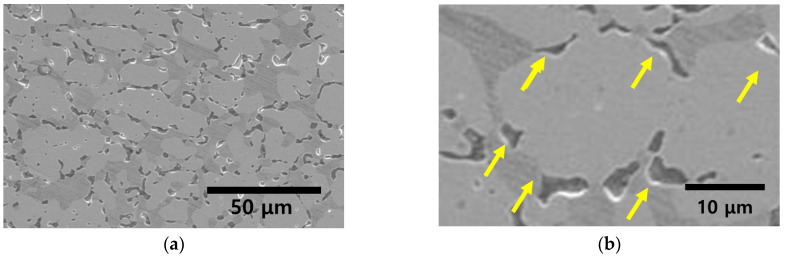

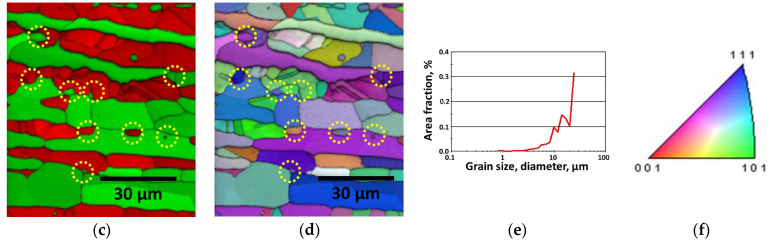
Microstructure image after heat treatment at 700 °C of super duplex stainless steel UNS S 32750: (**a**) magnification ×200, (**b**) magnification ×5000 (yellow arrow: secondary phase), (**c**) Phase-IQ (red: austenite, green: ferrite) after solution annealing at 1100 °C (yellow circle: secondary phase), (**d**) IPF-IQ image after solution annealing at 1100 °C (yellow circle: secondary phase), (**e**) grain size and area fraction, and (**f**) IPF color palette.

**Figure 5:**
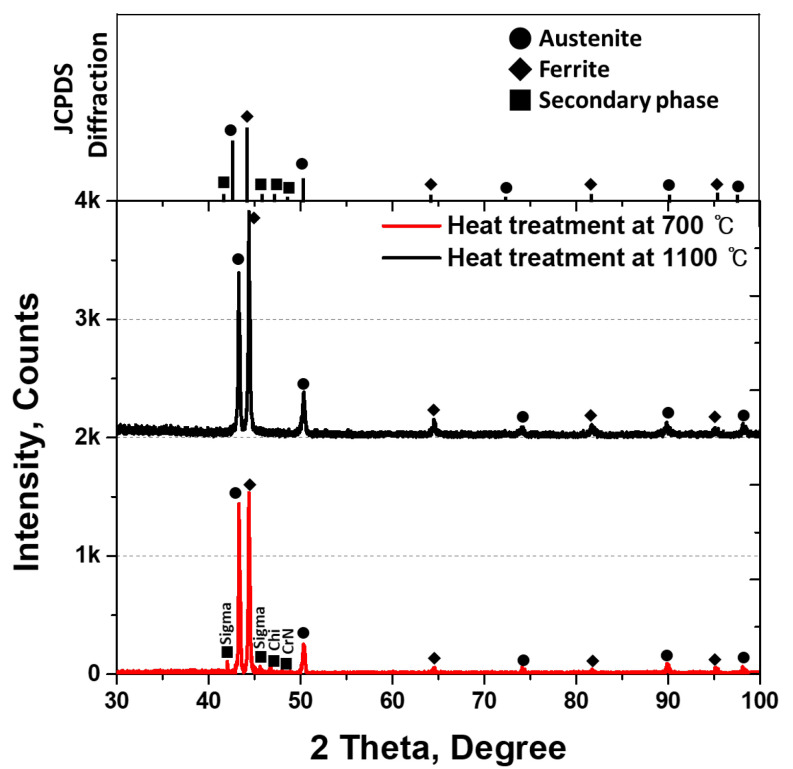
Intensity (counts) vs. 2θ (degrees) curve; X-ray diffraction pattern after heat treatment at 700 °C and 1100 °C of super duplex stainless steel UNS S 32750.

**Figure 6:**
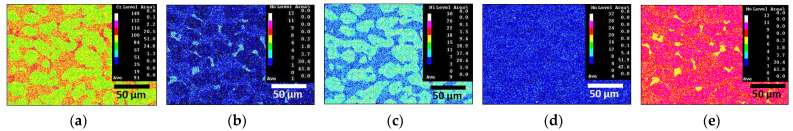
Mapping image of chemical composition by electron-probe microanalysis after heat treatment at 700 °C of super duplex stainless steel UNS S 32750: (**a**) Cr, (**b**), Mo, (**c**), Ni, (**d**) Mn, and (**e**) Fe.

After heat treatment at 700 °C, secondary-phase precipitation occurred, as depicted in Figure 4, illustrating the microstructure of the secondary phase. Precipitation of the secondary phase was initiated at the austenite boundaries. The precipitated secondary phase grew along the boundaries, which was favorable for phase transformation. The secondary phase was distinguished by bright and etched regions. The differences in the secondary phases were analyzed using XRD, EPMA, and EDS, as shown in Figure 5 and Figure 6, as well as in Table 3 [16,32].

EBSD analysis revealed that the precipitated secondary phases were two distinct phases, BCC and FCC, both finer than 10 μm. These secondary phases appeared to have crystallographic orientations similar to the surrounding matrix. They were observed to grow along the grain boundaries of ferrite or austenite during growth, maintaining similar crystal orientations to the existing microstructure.

XRD intensity was measured to identify the phases. The XRD analysis revealed that the secondary phase comprised Sigma, Chi, and CrN, with peaks at 42°, 46°, and 47°, respectively. The peaks of the secondary phase exhibited a low intensity, which was attributed to the irregular morphology of the secondary phase grown along the boundaries (the decreased diffraction angle of the X-rays).

The secondary phase was precipitated along the boundaries between the austenite and ferrite. These boundaries facilitated the phase transformation owing to the altered lattice structure, making nucleation along the boundaries easier. Along the boundaries, Sigma developed, and beneath Sigma, the Chi phase was formed.

### 3.3. Chemical Composition of Secondary Phase

The chemical composition of the secondary phase of UNS S 32750 was determined using EPMA and EDS. The EPMA results are shown in Figure 6, and the EDS results are presented in Table 3 [16,35]. The alloy distribution was assessed via EPMA, and the constituents of each phase were determined using EDS. In the EPMA analysis, direct evidence of Cr and Mo was observed in the secondary phase, whereas Fe and Ni were deficient. Within the secondary phase, the Sigma phase exhibited a high corrosion resistance with Cr (31.0 wt%) and Mo (9.1 wt%). Surrounding the Sigma phase, the etched areas revealed Chi with lower chemical compositions of Cr (22.0 wt%) and Mo (2.1 wt%). These findings are consistent with the existing literature [16,29]. The sigma phase is Cr-rich, while the chi phase is Cr-deficient. This suggests that the precipitation of secondary phases is more influenced by compositional factors than by changes in the lattice structure.

The secondary phase, Sigma (43°, 45°), initiated precipitation owing to the co-precipitation of the major alloying elements, Cr and Mo, during the transformation from ferrite to austenite [32,38]. Subsequently, Chi (47°) grew because of deficiencies in Cr and Mo in the surrounding area, whereas unalloyed N formed CrN (48°). The precipitation of the secondary phase began because of the changes in the chemical composition resulting from the phase transformation, thereby influencing the formation of the passivation layer. This precipitation behavior of the secondary phase was consistent with that in the existing literature.

### 3.4. Electrochemical Behavior

**Figure 7:**
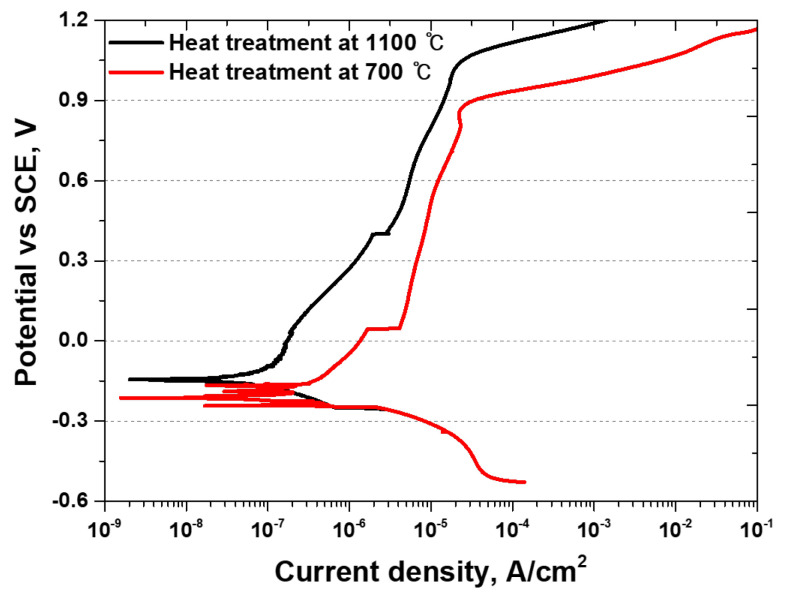
Potential (V) vs. current density curve (A/cm^2^); potentiodynamic polarization curve of super duplex stainless steel UNS S 32750 in 3.5 wt% Nacl electrolyte solution.

The impact of secondary phase precipitation on corrosion was analyzed using potentiodynamic polarization tests, with the results presented in Figure 7 and Table 4. AISI 2507 exhibited a high potential (E_corr_) and low corrosion current density (I_corr_) in the passive region during activation polarization. The pitting potential (E_pit_) was recorded at 1020 mV, consistent with the corrosion behavior of stainless steel reported in the literature. After the precipitation of secondary phases, a double-loop behavior was observed during activation polarization, which aligns with the potential differences between the secondary phases and the base structure. After active polarization, the passivation behavior showed a decreased pitting potential compared to solution heat-treated SDSS.

The potentiodynamic polarization tests indicated that the secondary phases weakened the passivation layer. Compositional segregation weakened the passivation layer and accelerated its breakdown, reducing the pitting potential from 1040 mV to 890 mV. The secondary phases facilitated the corrosion of UNS S 32750, increasing uniform corrosion rates and accelerating passivation film degradation. These phases acted as sites for pitting corrosion, reducing the overall corrosion resistance of UNS S 32750.

### 3.5. Discussion

The secondary phase precipitated at the grain boundaries of austenite owing to the saturation of Cr and Mo at the grain boundaries, which was facilitated by the growth of austenite. Thereafter, it exhibited growth behavior of the secondary phase along the grain boundaries of austenite. Initially, the precipitated secondary phase, Sigma, had a high composition of Cr and Mo, owing to saturation [34,39]. During the growth of the Sigma phase, deficiencies in Cr and Mo occurred, leading to the formation of the Chi phase. Unalloyed N, which was not incorporated into Sigma and Chi, formed CrN at its periphery [16,35,46]. Consequently, the resulting secondary phase, along with the austenite and ferrite, differed in chemical composition from the major alloying elements. This disparity in alloying elements contributed to variations in the galvanic potential, thereby reducing the corrosion resistance.

**Figure 8:**
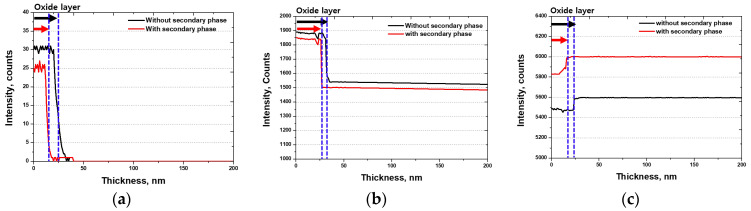
Intensity (counts) vs. thickness (nm) curve; glow-discharge spectrometer (GDS) results with or without secondary phase of super duplex stainless steel UNS S 32750: (**a**) GDS results of O, (**b**) GDS results of Cr, and (**c**) GDS results of Fe (black and red arrows, passivation layer thickness).

The corrosion resistance of the stainless steel was maintained by a passivation layer (Cr_2_O_3_), as determined by the PREN [18,47]. Although the strengthening factors of the passivation layer are well documented in the existing literature, corrosion resistance in the presence of secondary phases is less understood. The precipitation of secondary phases is known to form Cr-depleted zones, which are detrimental to corrosion resistance. However, research on the thickness of the passivation layer and the O concentration under different processing conditions is scarce. Therefore, the surface was polished with colloidal silica (surface roughness < 5 nm), cleaned with ethanol, and analyzed for the intensities of O, Cr, and Fe, which are the major alloying elements, using GDS. The results are depicted in Figure 8, showing a decrease in the intensities of O and Cr and an increase in the intensity of Fe. Considering the intensity of GDS, the depth of the O layer corresponded to 25 nm, indicating the thickness of the passivation layer. However, after the precipitation of the secondary phases, the depth of the O layer decreased from 25 nm to 20 nm [48,49]. The precipitation of the secondary phases may reduce the thickness of the passivation layer, potentially decreasing the corrosion resistance. Considering the lattice structure, chemical composition, and GDS results, the Cr segregation caused by secondary phases contributed to the thinning of the passivation layer.

**Figure 9:**
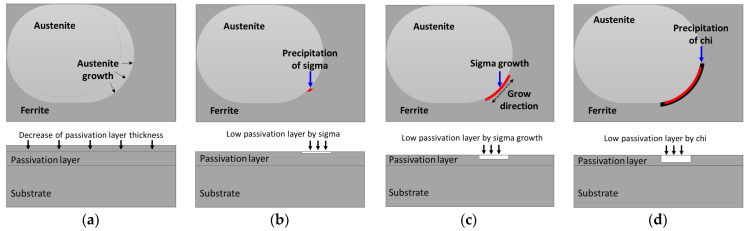
Schematic of passivation-layer formation with or without secondary phase of super duplex stainless steel UNS S 32750: (**a**) austenite growth and decrease in passivation layer (black arrow, austenite growth direction), (**b**) precipitation of secondary phase and decrease in partial passivation layer thickness, (**c**) growth of Sigma and decreased passivation-layer thickness, and (**d**) precipitation Chi phase and lower passivation-layer thickness.

The precipitation of secondary phases weakens the passivation layer by forming a Cr-defected zone owing to the segregation of the major alloy [29]. However, the formation of secondary phases decreased the thickness of the passivation layer via alloy segregation. Therefore, alloy segregation acts as a factor that reduces the thickness of the passivation layer; the corresponding schematic is shown in Figure 8. The precipitation of secondary phases led to differences in the thickness of the oxide layer formed on the surface, potentially causing variations in the electrochemical properties of SDSS UNS S 32750. The precipitation of the secondary phase decreases the thickness of the passivation layer due to segregation of the major alloys, which decreased the corrosion resistance. Figure 9 shows a schematic diagram of secondary phase precipitation, growth, and thickness decrease of the passivation layer.

**Figure 10:**
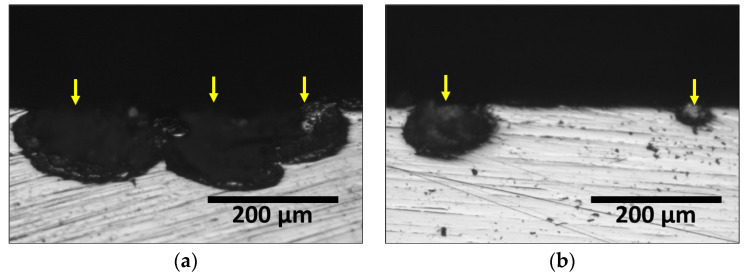
Cross-section image after potentiodynamic polarization curve of super duplex stainless steel UNS S 32750 in 3.5 wt% NaCl electrolyte solution (yellow arrow: pitting morphology): (**a**) heat treatment at 700 °C and (**b**) heat treatment at 1100 °C.

Consequently, the presence of secondary phases decreased corrosion resistance, as confirmed by the potentiodynamic polarization tests. The secondary phases increased the corrosion rate and decreased corrosion resistance. Stainless steel corrosion typically grows in the form of pitting, as shown in Figure 10, illustrating the corrosion morphology of UNS S 32750. The corrosion morphology varied with the corrosion rate; pits coalesced into larger forms as corrosion progressed. Secondary phases accelerated pit growth and promoted corrosion at multiple sites, acting as corrosion initiation points due to the weakened passivation layer.

## 4. Conclusions

To apply SDSS UNS S 32750 as a material for Li-ion battery cases, the passivation layer was analyzed. The following conclusions were drawn:

(1) SDSS SAF2507 exhibited precipitation of the secondary phase at 700 °C. The precipitation of the secondary phase began at the boundaries of austenite when ferrite transformed into austenite. The ferrite had a high Cr content of 26.6 wt%, whereas austenite had a lower Cr content of 23.3%. As a result, during the transformation from ferrite to austenite, over 3% of the Cr from ferrite was transferred to austenite. Owing to the precipitation of undissolved Cr and Mo at the austenite boundaries, the secondary phase also undergoes precipitation. The precipitated secondary phase grew along the austenite boundaries.
(2) The secondary phase, Sigma, precipitated primarily because of the segregation of Cr and Mo, was followed by the formation of the Chi phase because of deficiencies in Cr and Mo. Additionally, N, which remains unincorporated into Sigma and Chi, precipitated as CrN. The precipitation of the secondary phase, which was influenced by the segregation of Cr and Mo, led to differences in the PREN. These differences in the PREN contributed to galvanic corrosion and reduced corrosion resistance. The galvanic corrosion resulted from compositional variations. Therefore, if ignition occurs and the secondary phase precipitates during the use of Li-ion batteries, this may lead to a decrease in corrosion resistance due to galvanic corrosion.
(3) The secondary phase affected the thickness and chemical composition of the passivation layer. After the solution heat treatment, the depth of the passivation layer was 30 nm, whereas after the precipitation of the secondary phase, it decreased to 25 nm. The precipitation of Cr in the secondary phase decreased the thickness of the passivation layer. This decrease in the thickness of the passivation layer reduces the corrosion resistance and may shorten the lifespan of Li-ion batteries. Therefore, applying SDSS UNS S 32750 as a material for Li-ion battery cases can enhance stability. However, if recycled, it must undergo solution heat treatment without failure because of the secondary phase.

## Figures and Tables

**Table 1:** Chemical composition of super duplex stainless steel UNS S 32750 BY ICP-MS for advanced Li-ion battery case material.

Elements	C	N	Mn	Ni	Cr	Mo	Fe	PREN
Chemical composition (wt%)	0.01	0.27	0.8	6.8	25.0	3.8	Bal	42.0

**Table 2:** Chemical composition of austenite and ferrite with manufacturing process (casting and solution annealing) on super duplex stainless steel UNS S 32750 obtained via EDS.

	Phase	C	N	Mn	Ni	Cr	Mo	Fe	PREN
Casted	Austenite	0.01	0.55	1.1 ± 0.2	7.8 ± 0.5	22.8 ± 1.1	3.0 ± 0.4	Bal	41.4
	Ferrite	0.01	0.05	0.8 ± 0.2	5.6 ± 0.5	27.2 ± 1.1	4.5 ± 0.4	Bal	42.9
Annealed	Austenite	0.01	0.51	1.1 ± 0.2	7.9 ± 0.5	23.3 ± 1.2	3.2 ± 0.4	Bal	42.0
	Ferrite	0.01	0.05	0.8 ± 0.2	5.5 ± 0.5	26.6 ± 1.2	4.4 ± 0.4	Bal	42.1

**Table 3:** Chemical composition of the precipitated secondary phase (Sigma and Chi) on super duplex stainless steel UNS S 32750.

Phase	Cr	Mo	Ni	Mn	Fe	PREN
Sigma	31.0 ± 1.4	9.1 ± 1.1	4.5 ± 0.8	0.5 ± 0.1	56.3 ± 3.1	61.0
Chi	22.0 ± 0.9	2.1 ± 0.5	9.5 ± 1.2	1.0 ± 0.2	65.2 ± 2.4	28.9

**Table 4:** Major value of potentiodynamic polarization curve after heat treatment at 700 °C and 1100 °C of super duplex stainless steel UNS S 32750 in 3.5 wt% NaCl electrolyte solution.

Heat Treatment	E_corr_	I_corr_	E_pit_
At 700 °C	−270 mV	2 × 10^−7^ A/cm^2^	890 mV
At 1100 °C	−150 mV	1 × 10^−9^ A/cm^2^	1040 mV

## Data Availability

The original contributions presented in the study are included in the article, and further inquiries can be directed to the corresponding authors.
